# Intimo-Intimal Intussusception due to Stanford Type A Acute Aortic Dissection Presenting as Cerebral Infarction

**DOI:** 10.1155/2022/6258504

**Published:** 2022-02-01

**Authors:** Takanori Kono, Takahiro Shojima, Tomoyuki Anegawa, Hiroyuki Otsuka, Eiki Tayama

**Affiliations:** Division of Cardiovascular Surgery, Department of Surgery, Kurume University School of Medicine, Kurume, Fukuoka, Japan, 67 Asahi-machi, Kurume City, Fukuoka 830-0011, Japan

## Abstract

Complete circumferential dissection is a rare clinical presentation of aortic dissection, wherein the dissected flap has the potential to cause intimo-intimal intussusception, which can lead to several catastrophic complications. We report a case of Stanford type A acute aortic dissection with intimo-intimal intussusception causing unstable cerebral ischemic symptoms. An 82-year-old man was taken to another hospital with severe intermittent dizziness. Head magnetic resonance imaging revealed multiple right-hemispheric cerebral infarctions. Computed tomography also showed a “missing flap,” indicating that the intimal flap was observed in the aortic root and arch but not in the ascending aorta. The patient was referred to our hospital for emergent surgery. Intraoperatively, the intimal tear was found to be circumferential, and the transected intima was folded and superimposed from the origin of the brachiocephalic artery to the aortic arch. Ascending aortic replacement and aortic valve replacement were performed; the postoperative course was good.

## 1. Introduction

Circumferential dissection with intimo-intimal intussusception is a rare clinical presentation of aortic dissection. Depending on where the intima invaginates, intimal intussusception causes dynamic obstruction of various arteries. Herein, we report a case of Stanford type A acute aortic dissection with intimo-intimal intussusception causing unstable cerebral ischemic symptoms.

## 2. Case Report

**Figure 1:**
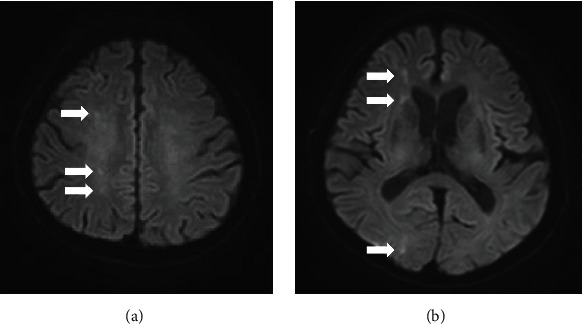
Head diffusion-weighted magnetic resonance images show acute multiple right-hemispheric cerebral infarctions (white arrows).

**Figure 2:**
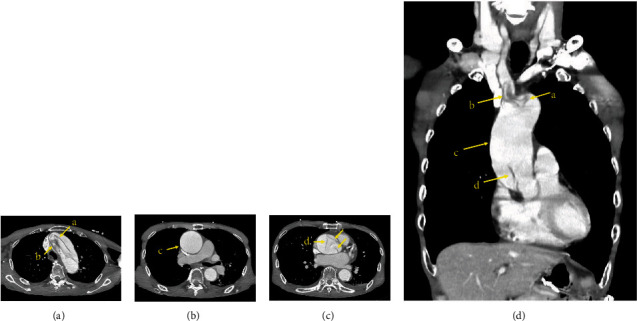
Preoperative contrast-enhanced computed tomography. Multiple intimal flaps were present in the aortic root (arrow D in c) and arch (arrows A and B in a) but not in the dilated ascending aorta (arrow C in b), thus constituting a missing flap. A reconstructed coronal image shows discontinuity of the intimal layer in the ascending aorta (arrow C in d) and the intimal flap invaginating the brachiocephalic artery (arrow B in d).

**Figure 3:**
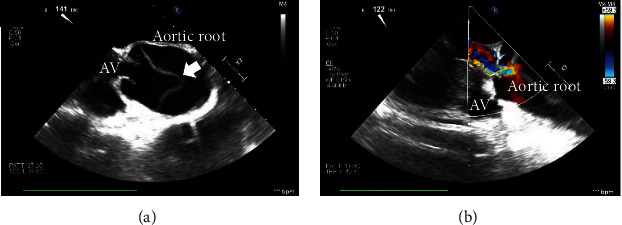
Transesophageal echocardiography shows the intimal flap (white arrow) at the aortic root (a) and aortic regurgitation (b).

An 82-year-old man was transferred to another hospital with severe dizziness with repeated exacerbations and remissions. Head magnetic resonance imaging revealed multiple right-hemispheric cerebral infarctions (Figures 1(a) and 1(b)). Plain computed tomography (CT) indicated acute aortic dissection. He was transferred to our hospital for emergent surgery. His blood pressure was 148/77 mmHg, with no difference between the right and left measurements. His heart rate was 102 beats/min. A grade II/VI “to and fro” murmur was audible along the left sternal border. The arteries of the lower extremities were palpable, and no murmurs were heard in the cervical vessels. Contrast-enhanced CT revealed the presence of an intimal flap in the aortic root (Figure 2(a)) and arch (Figure 2(b)) but not in the ascending aorta, termed as “missing flap” (Figure 2(c)). The intimal flap invaginated the brachiocephalic artery (Figure 2(d)). Preoperative transoesophageal echocardiography (TEE) showed the intimal flap in the aortic root and moderate aortic regurgitation (AR) (Figure 3). These findings were compatible with Stanford type A acute aortic dissection; emergency surgery was performed. Intraoperatively, cardiopulmonary bypass (CPB) was established from the superior and inferior vena cava to the right axillary and femoral arteries. Upon examining the ascending aorta under circulatory arrest with a bladder temperature of 25°C, the intimal tear was found to be circumferential; the transected intima was folded and superimposed on the aortic wall from the brachiocephalic artery origin to the aortic arch. The intima was withdrawn proximally, and no additional aortic arch tears were observed. Ascending aorta replacement using a 28 mm J graft with one branch (Japan Lifeline Co., Ltd., Tokyo, Japan) and suspension of the commissure of the aortic valve were performed. The stump of the aorta was reinforced with the felt sandwich technique for the proximal and distal anastomoses, and BioGlue (CryoLife, Inc., GA, USA) was used for proximal anastomosis. During weaning from CPB, TEE revealed moderate AR. The second aortic clamping was performed, and aortic valve replacement with a 21 mm Inspiris Resilia biorosthetic valve (Edwards Lifesciences LLC, Irvine, USA) was undertaken. The postoperative course was good without any neurological complications.

## 3. Discussion

Complete circumferential dissection of the aorta and inversion of the intimal flap is an extremely rare condition first reported by Bostroen in 1887 [1]. Chiari described it as “inversion of the inner cylinder” in 1909, and Hufnagel named it “intimo-intimal intussusception” in 1962 [2]. CT is a rapid and useful imaging modality for diagnosis; TEE is also reported to be effective for cases difficult to diagnose [3]. The “missing flap” in this case is a characteristic finding of circumferential aortic dissection [4]. Furthermore, if multiple complicated flaps are found distal to the missing flap, circumferential aortic dissection should be strongly suspected. Intimal intussusception can cause dynamic obstruction of various vessels. On the distal side, hemodynamic deterioration or clinical features of pseudocoarctation can occur when it is situated in the descending aorta, and neurological disorders can occur if the arch vessels are occluded [5]. On the proximal side, acute AR due to the detachment of the aortic valve commissure and myocardial ischemia due to the blockage of the coronary arteries can occur [5]. In our case, dizziness without any chest pain was the main complaint. Aortic dissection requires emergency surgery, but it is sometimes difficult to diagnose if the patient has neurological symptoms. Neurologists and cerebrovascular physicians are the initial responders to patients presenting with neurological symptoms in the absence of chest and back pains; they are less likely to suspect acute aortic dissection. It is important to recognize that aortic dissection may be masked by stroke symptoms because intravenous administration of recombinant tissue plasminogen activator is contraindicated in patients with brain infarction and aortic dissection. In this case, the cause of cerebral infarction is difficult to determine, but it is most likely due to microemboli. The possibility of unstable blood flow, microthrombus between the intima caused by unstable blood flow, and microembolization of the aortic intimal fragments were considered. No thrombus was observed on the invaginated intimal flap by visual inspection in the surgical field.

In conclusion, although circumferential Stanford type A acute aortic dissection with intimo-intimal intussusception is a rare condition and sometimes difficult to diagnose, it is important to understand the imaging findings and pathophysiology of circumferential dissection to establish the appropriate diagnosis and initiate surgery.

## Data Availability

The data that support the findings of this study are available from the corresponding author, TK, upon reasonable request.

## Supplementary Materials

Supplementary Materials Video 1: operative findings. The descriptions are presented in the video. Ao: aorta; AscAo: ascending aorta; AR: aortic regurgitation; AVR: aortic valve replacement; Ax: axillary; BCA: brachiocephalic artery; CPB: cardiopulmonary bypass; FA: femoral artery; IVC: inferior vena cava; LCC: left coronary cusp; NCC: noncoronary cusp; Rt: right; SVC: superior vena cava.
